# The impact of immigration detention on mental health: an updated systematic review

**DOI:** 10.1186/s12888-026-08149-z

**Published:** 2026-05-16

**Authors:** Reem Khalid Saifeldeen, Sasha Menon, Naomi Glover, Cornelius Katona

**Affiliations:** 1 Mental Health Service, Hamad Medical Corporation, P.O Box 3050, Doha, Qatar; 2 Department of Psychiatry, University of Cambridge, Cambridge, UK; 3 Division of Psychiatry, University College London, 6th Floor, Wing A, Maple House, 149 Tottenham Ct Rd, London, W1T 7NF UK; 4 Helen Bamber Foundation, 26 Westland Place, London, N1 7JH UK

**Keywords:** Immigration, Detention, Asylum seeker, Refugee, Mental health, Migrant

## Abstract

**Background:**

The number of refugees and people seeking asylum worldwide has risen significantly over the past 10 years. These individuals are often placed in immigration detention facilities following their migration journey. Previous studies, including a systematic review conducted in 2018, indicate that detention may be associated with an increase in mental health difficulties. The present review aimed to update the 2018 review by reporting on more recent studies investigating the prevalence of mental disorders and symptoms in immigration detainees, and whether the length of detention increases the likelihood of such disorders.

**Methods:**

Six databases were searched for quantitative, peer-reviewed studies reporting on the prevalence of psychiatric symptoms or disorders in adults and children who had experienced immigration detention. The quality of included studies was assessed using the Joanna Briggs Institute Critical Appraisal Checklist for Studies Reporting Prevalence Data. The findings were presented narratively.

**Results:**

Fourteen studies were included, reporting on a total of 2777 participants. The prevalence of significant mental health problems was high in both adults and children subjected to immigration detention. The most common disorders reported were post-traumatic stress disorder (PTSD), anxiety, and depression. Children also experienced high levels of emotional dysregulation and behavioural symptoms. In adults, longer durations of detention were consistently associated with a higher likelihood of experiencing mental health difficulties. Factors related to detention environments were also associated with the prevalence of psychological symptoms and diagnosable mental health conditions.

**Conclusions:**

Overall, this review confirms that immigration detention has negative mental health consequences across a variety of immigration detention facilities and detainee characteristics. Recommendations based on findings are presented, including eliminating child detention, imposing upper limits to the duration of adult detention, improving mental health screening pre-detention, and providing evidence-based psychological care during detention.

**PROSPERO registration number:**

CRD42023487205.

## Introduction

### Background

At the end of 2024, the United Nations High Commissioner for Refugees (UNHCR) estimated that there were 42.7 million refugees and 8.4 million asylum-seekers globally [1]. Over the past decade, the number of displaced individuals worldwide has doubled [2]. In the United Kingdom (UK) alone, asylum applications rose sharply between 2021 and 2024, reaching a record 84,231, and then increased by a further 17% by June 2025 [3]. Asylum-seekers are frequently held in immigration detention facilities, with over 800 immigration detention centres currently in use around the globe [4]. Within the European Union, over 100,000 people are currently in detention for immigration purposes [5]. This detention primarily takes place during the periods while a person’s asylum claims are being processed, when their identities are being verified, or while awaiting deportation or removal [5].

The UNHCR Detention Guidelines highlight that detention must be used as a last resort and be proportionate [6]. However, in practise these guidelines are not always applied [7]. For example, whilst they prohibit prolonged or indefinite detention, countries such as the UK, Australia, the United States of America (USA), and Canada have no upper limits to the length of time an individual may be detained [8–11]. In the UK, between 2019 and 2022, migrants spent between one day to more than four years in immigration detention, with 10.9% detained for six months or longer [12]. Furthermore, the conditions of immigration detention centres are frequently substandard, contributing to declines in detainees’ overall wellbeing [13]. Reports have highlighted poor access to physical and mental health care, overcrowding, disparaging behaviour from staff, unsanitary environments, poor nutrition, and limited to no access to outdoor spaces [13–15].

The lack of consistency in following the recommended detention guidelines is further demonstrated in the continued detention of children for immigration purposes, which the UNHCR explicitly prohibits [7]. Detained children have been found to experience numerous health-related harms [16], such as mental and physical disorders, potential malnutrition, susceptibility to illnesses, and developmental concerns [17]. Despite this, 77 countries are known to detain children based on their migratory status [18]. For example, Australia exercises mandatory detention for all children who arrive without valid immigration documentation [10]. Worldwide, there are at least 330,000 children held in immigration detention per annum [18].

### Asylum-seeker and refugee mental health

Refugees and asylum-seekers are vulnerable to mental health challenges due to facing adversity in their countries of origin, during their migration journey, and throughout the resettlement process in their countries of arrival [19]. The development of mental disorders can be influenced by premigration experiences, including experiencing interpersonal and external trauma [20]. Such experiences include childhood personal traumatic events (PTEs), such as familial and external violence [21], as well as exposure to torture, war experiences, and human rights violations [22–24]. Migration journeys in themselves can be stressful experiences, possibly contributing to the development of mental health conditions [25]. Specific peri-migration stressors include the length of the migration journey, as well as exposure to trauma during transit [26]. Post-migration stressors are significantly associated with post-traumatic stress disorder (PTSD), anxiety, and depression [27]. These stressors include having temporary legal status, experiencing discrimination, and acculturative stress [27–29].

This population exhibits consistently high rates of mental disorders, highlighted in meta-analyses showing prevalences of PTSD and depression, respectively, to be between 31% and 32% [30, 31]. Additionally, the prevalence of psychosis is indicated to be between 1% and 2% [30, 31]. The prevalence of anxiety is high compared to the general population, ranging from 11% to 13% for diagnosed anxiety disorders, and up to 52% for self-reported anxiety symptoms [30, 32, 33]. The increased prevalence of these mental disorders has been found regardless of the area to which migrants are relocated and possibly persist irrespective of the length of residence in the country of arrival [32].

### Immigration detention and mental health

Individuals detained for migration related purposes identify detention as a dehumanising experience, involving deprivation, isolation, and the fracturing of relationships [34]. In a report by Human Rights Watch, immigration detainees consistently expressed the detrimental effects of detention on their well-being [35]. Previous literature has identified high levels of mental health disorders in detainees, most commonly depression, anxiety, and PTSD [36, 37]. Additionally, detention appears to increase the incidence of self-harming behaviours [38, 39]. Prolonged detention may be associated with more severe mental difficulties, persisting beyond release from detention [40]. A UK report found that detainees who experienced indefinite detention with no set release date experienced high levels of stress, due to lack of knowledge about the duration of their detention inhibiting their ability to mentally adapt [8]. Detainees note that waiting for indefinite periods of time is disempowering, emphasising a prolonged lack of control over decisions impacting their futures [41].

A previous systematic review by von Werthern et al. [42] exploring the impact of immigration detention on mental health included twenty-six studies: these involved child and adolescent - as well as adult detainee populations. The review found that anxiety, PTSD, and depression were the most reported disorders. Detainees had more severe mental symptoms compared to non-detainees. Both detention duration and greater exposure to pre-migration trauma were positively associated with higher symptom severity.

In the seven years since this review was published, the number of asylum-seekers worldwide has increased by 138.5% [1]. Additionally, there has been a growing body of evidence on this topic which has not yet been systematically reviewed. There was therefore a need to update this review with current research to identify if evidence of immigration detention exacerbating mental difficulties has remained consistent, or whether the evidence prior to 2018 has led to reforms reducing such adverse effects. This also provided the opportunity to determine whether there was further evidence regarding the relationship between duration of detention and mental health problems.

### Current review

The present review updates the systematic review by von Werthern and colleagues [42], examining more recent evidence on the impact of immigration detention on the mental health of detainees, and the relationship between prolonged detention and detainee mental health. The primary objective of this review was to report the prevalence of mental health symptoms and disorders in individuals who have experienced immigration detention. The secondary objectives were to explore the relationship between detention duration and mental health symptoms and disorders in detainees, as well as identifying whether any sub-groups are particularly vulnerable to the development of these symptoms. The research questions were as follows:

1. What is the prevalence of mental health symptoms and disorders experienced by immigration detainees?
2. What is the effect of prolonged detention on the mental health of immigration detainees?

## Methods

This systematic review was conducted and reported in accordance with the Preferred Reporting Items for Systematic Reviews and Meta-Analyses (PRISMA) 2020 guidelines [43]. The review protocol was registered with PROSPERO (registration number: CRD42023487205).

### Inclusion and exclusion criteria

Studies were included if they (a) included samples of adult or child asylum-seekers, refugees, or migrants who had previously or were currently experiencing detention for immigration related purposes (b) reported the prevalence of mental health disorders or symptoms in the sample, and/or reported on the relationship between the duration of detention and mental health disorders or symptoms (c) were peer-reviewed and (d) were quantitative in nature. Additional restrictions were placed on the dates of publication (only including papers published between 2017 and 2025), to avoid overlap with the 2018 systematic review and to reflect contemporary detention practises. The inclusion date of 2017 was chosen to capture studies potentially missed prior to publication of the previous review. Only studies in the English language were included due to unavailability of translation support. No restrictions were placed on the type of quantitative study design, country of study, detainees’ country of origin, or the age of detainees. Due to the limited and heterogenous evidence base, studies employing self-report, screening, and diagnostic measures were included to maximise inclusivity and ensure a comprehensive synthesis of available findings.

Qualitative studies, reviews, letters, grey literature, commentaries, books, editorials, conference abstracts, and dissertations were excluded. Studies with samples of detainees who were not held in official immigration detention or removal centres (e.g. reception centres with freedom of movement, refugee camps, prisons) or in community detention were also excluded. Furthermore, studies which only provided aggregated data for detainees and non-detained individuals were excluded. If disaggregated data was not reported, authors were contacted for this data. Studies were excluded if no response was received or if disaggregated data was unavailable. Finally, studies identified in the search, but which were included in the previous 2018 review were excluded.

### Search strategy and screening

Relevant studies were identified through searching Ovid MEDLINE, PsycInfo, Embase, CINAHL Plus, Web of Science, and PTSDpubs (from inception to 15/05/2025), restricting results to English-language publications from 2017 onwards. Citation searches of excluded systematic reviews relevant to the topic were conducted to identify potential studies for inclusion. Search strings were developed based on the Population, Intervention, Comparison, Outcome (PICO) framework [44]. No search string was developed for the ‘comparison’ PICO element, as this would limit the scope of the review and exclude valuable evidence. However, studies with comparison groups were still included. The remaining three PICO components were adapted into respective search strings: population characteristics (asylum-seekers and refugees), intervention (immigration detention), and outcomes (mental health symptoms and disorders) (Table 1).

**Table 1:** Breakdown of search strategy for Ovid databases ^**a**^

PICO Element	Search String (Ovid – MEDLINE, Embase, PsycInfo)
Population	(asylum adj1 seek*) OR asylumseeker* or asylum-seeker* OR asylum applicant* OR (asylum adj1 claim*) OR refuge* or migrant* or immigrant*
Intervention	detention OR (depriv* adj2 liberty) OR detain* OR imprison* OR incarcerat* OR (reception adj1 cent*) OR asylum adj1 cent* OR (accomodation adj1 cent*) OR temporary protection OR custod* OR prison* or jail*
Outcome	mental health OR mental ill* OR mental disorder* OR psychiatric disorder* OR psychiatric diagnos* OR behavio? r disorder* OR mental well* OR psychological wellbeing OR psychiatric symptoms OR psychological status OR anxi* OR depress* OR stress* OR trauma* OR post-traumatic stress OR PTSD OR CPTSD OR intrusi* OR re-experienc* OR disorders of extreme stress OR psychosis OR psychotic OR delusion* OR hallucinat* OR paranoi* OR delusion* OR mood disorder* OR bipolar OR mania OR manic OR hypomani* OR mood swing* OR neurodevelopment* OR autis* OR ASD OR ASC OR ADHD OR attention-deficit hyperactivity disorder OR learning disabilit* OR learning difficult* OR intellectual disabilit*

^a^ Proximity, truncation, and wildcard operators varied in non-Ovid databases (based on database specifications)

References from each database were exported to Covidence [45] for review, where duplicates were identified and excluded. Study titles, abstracts, and full texts were screened by the first reviewer (RS) who screened all records, and independently by the second reviewer (SM) who screened 10% of the total number. Disagreements about study inclusion were resolved through discussions between reviewers, and if an agreement was not reached, a third reviewer assisted (CK, NG). At both title, abstract, and full-text screening stage, 100% inter-rater agreement was achieved, which was regarded as highly satisfactory. Two full texts did not specifically present prevalences, therefore the respective authors were contacted to request this data. As no response was received for either study, they were excluded from this review.

### Data extraction and synthesis

The following information was extracted from each included study: author/year, aim, sample, sampling method, comparison group (where applicable), sociodemographic information, country of origin, country of study, detention type, length of detention, mental health outcomes and measurement tools, response rate, prevalence data, associated factors, subgroup differences, and data on the impact of detention duration.

Due to the heterogeneity in study design and outcome measures, a narrative synthesis was undertaken, guided by recommendations from Popay et al., [46]. The variance in outcomes measures between studies restricted the ability to pool results, hence no meta-analysis was conducted. Results were organised thematically to aid interpretation. First, findings were grouped according to whether the study sample was comprised of adults or children/adolescents. Within the two respective sup-groups, results were further synthesised by symptom clusters and diagnostic categories (e.g. depression and anxiety, PTSD, neurodevelopmental disorders), as well results about the impact of detention duration. This approach allowed patterns of association to be identified across studies and to highlight areas of consistency and variance in the included studies.

### Quality appraisal

The quality of included studies was appraised independently by two reviewers using the Joanna Briggs Institute Critical Appraisal Checklist for Studies Reporting Prevalence Data [47]. The first reviewer (RS) assessed the quality of all included studies, and the second reviewer (SM) appraised the quality of a randomly selected subsample of 10%. Disagreements were resolved through discussions between the reviewers. 100% inter-rater agreement was achieved. The JBI prevalence checklist assesses the risk of bias and methodological quality in studies reporting prevalence data across nine items. For each study, items are rated as ‘Yes’, ‘No’, ‘Unclear’, or ‘Not Applicable’. The checklist does not produce a definitive numerical or categorical value of quality. Rather, item-level ratings are presented to inform findings. Studies were not excluded based on quality to maximise the comprehensiveness of the evidence base and to avoid introducing bias through selective exclusion. However, methodological limitations were considered when assessing the strength of the included studies.

## Results

### Included studies

**Fig. 1:**
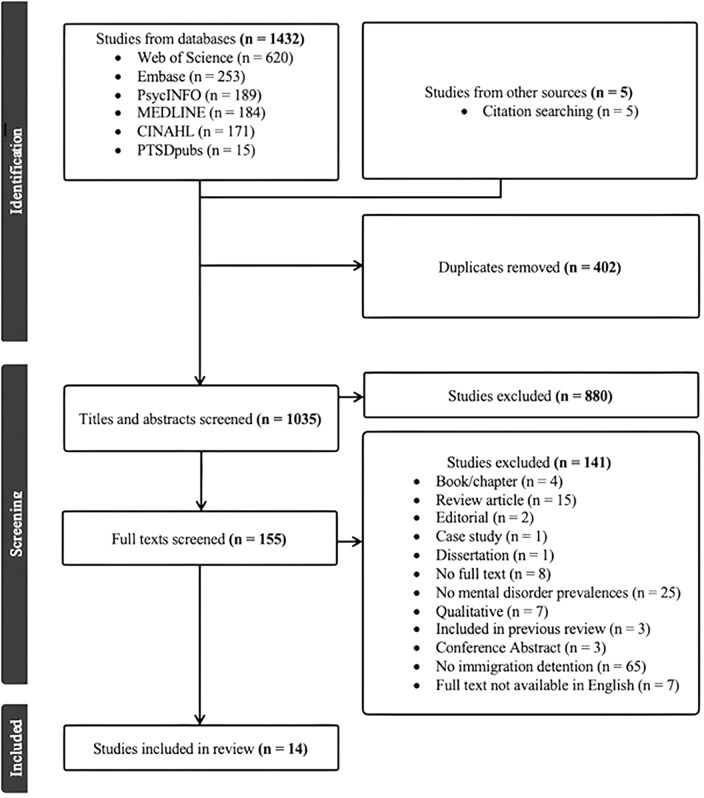
PRISMA 2020 flow diagram

Fourteen studies were included in the review (see Fig. 1), reporting on a total of 2777 participants. The studies were conducted in Mexico [48], Spain [49], USA [50–56], Portugal [57], UK [58], and Australia [59–61]. Of the included studies, six included adult participants [48, 49, 52–54, 57], and eight included children/adolescents [50, 51, 55, 56, 58–61]. The quality of each study was assessed (Table 2). Key characteristics for adult studies can be found in Table 2A, and for child/adolescent studies in Table 2B. Half of the studies presented data collected during detention [48–50, 53, 57, 60, 61], and half included data collected post-detention [51, 52, 54–56, 58, 59], The reporting of participants regions/countries of origins varied between studies, with some not reporting this information and one study reporting as many as seventeen individual countries of origin [58].

**Table 2A:** Key characteristics of adult studies

Author & Year	Study Design	Country of Study	Sample	Age	Gender	Region of Origin ^a^	Mental Health Outcomes ^b^	Detention Setting
Bakley et al., 2023	Cross sectional	Mexico	*N* = 306, adults deported from US and released into Mexican deportation stations	Mean (SD) = 38.3 (10.5) Range not reported	Male = 281 (91.9%) Other genders not reported	Mexico (100%)	Diagnosed mental health conditions	Three Mexican border stations (Mexican Institute of Migration) – Tijuana, Matamoros, Ciudad Juárez
Paloma et al., 2025	Cross sectional	Spain	*N* = 87, adult migrants detained in Spain	Mean (SD) = 30.92 (8.55) Range not reported	Male = 83 (95.4%) Female = 4 (4.6%)	Morocco (57.5%), Columbia (13.6%), Latin American/African (28.9%)	Depression, anxiety, self-harm in detention	Three Centres of Internment for Foreigners – Madrid, Valencia, Algeciras
Patler et al., 2025	Cross sectional	United States	*N* = 203, adult immigrants released from immigration detention between 2020–2021	Mean (SD) = 40.3 (10) Range not reported	Male = 178 (87.7%) Other genders not reported	Not reported	Diagnosed mental health disorders	US Immigration Customs Enforcement (ICE) facilities
Saadi et al., 2022	Cross sectional	United States	*N* = 493, detained adults (members of Rodriguez vs. Robbins class action litigation)	Mean (SD) = 37.1 (9,2) Range = 18.6–68.9	Male = 91.7% Other genders not reported	Not reported	Diagnosed mental health conditions	Four immigration prisons in Central Federal Court District of California
Saadi et al., 2025	Cross sectional	United States	*N* = 200, adults previously held in detention then released into US Sub-sample detention < 6 months = 92 Sub-sample detention > 6 months = 108	Mean (SD) = 40.3 (10) Range not reported	Male = 175 (87.5%) Female = 25 (12.5%)	Not reported	Presence of mental illness, PTSD	US Immigration Customs Enforcement (ICE) facilities
Santos et al., 2018	Retrospective observational	Portugal	*N* = 396, adult undocumented migrants held in detention	Mean (SD) = 34 (10) Range not reported	Male = 334 (84%) Female = 84 (16%)	Countries: India (6.1%), Bangladesh (6.3%), Brazil (13.1%), Cape Verde (10.6%), Morocco (5.8%), Ukraine (8.1%), data not provided (50%) Continents: Africa (41%), Asia (27%), Europe (16%), South America (14%), other (1%) ^c^	Diagnosed mental and behavioural disorders	Temporary detention centre in Oporto

a As reported by study authors

b Mental health outcomes which contribute to prevalence data

c Percentages do not total to 100 due to rounding by authors

**Table 2B:** Key characteristics of child & adolescent studies

Author & Year	Study Design	Country of Study	Sample	Age	Gender	Region of Origin ^a^	Mental Health Outcomes ^b^	Detention Setting
Amarasena et al., 2023	Cross sectional	Australia	*N* = 62, children and young people detained in offshore detention between 2013–2019	Mean (SD) = 9 (5.7) Range = 3 months – 22 years	Male = 38 (61%) Other genders not reported	Iran (47%), Iraq (11%), Sri Lanka (10%), no data (5%)	Mental health/ Neurodevelopmental symptoms and diagnoses	Offshore detention centres on Nauru
Ehntholt et al., 2018	Cross sectional	United Kingdom	*N* = 35, adolescents/young people previously held in detention as unaccompanied asylum-seeking children	Mean (SD) = 19.1 (1.12) Range = 16–21	Male = 21 (60%) Female = 14 (40%)	Afghanistan (31%), DRC (9%), Iran (9%), Uganda (9%), China (6%), Ivory Coast (6%), Eritrea (3%), India (3%), Kosovo (3%), Malawi (3%), Mongolia (3%), Morocco (3%), Nigeria (3%), Sierra Leone (3%), Somalia (3%), Sri Lanka (3%), Vietnam (3%) ^c^	PTSD, Major Depressive Disorder	Detained by British authorities unlawfully under premise of being adults
MacLean et al., 2019	Cross sectional	United States	*N* = 425, children held in detention Sub-sample aged 9–17 = 150	Mean (SD) = 10 (4) Range = 4–17	Gender not reported	Honduras (50%), Guatemala (22%), El Salvador (23%), Nicaragua (2%), Mexico (2%), Cuba (1%), other (1%) ^c^	PTSD, emotional and behavioural difficulties	US Immigration Customs Enforcement (ICE) facilities
Min et al., 2020	Cross sectional	United States	*N* = 42, children previously detained with mothers (*N* = 42, answered questionnaires)	Mean (SD) = 6.79 (4.42) Range = 2–16	Male = 18 (42.86%) Female = 22 (52.38%) Not reported = 2 (4.76%)	Honduras (33.3%), El Salvador (47.6%), Guatemala (19%) ^c^	Emotional and behavioural difficulties	Family detention centres – Dilley or Karnes Detention Centre
Sidamon-Eristoff et al., 2022	Cross sectional	United States	*N* = 84, children previously held in detention (64 parents completed interviews)	Mean (SD) = 7.78 (4.18) Range = 1–17	Male = 38 (45.2%) Female = 46 (54.8%)	Honduras (53.6%), El Salvador (23.8%), Mexico (10.7%), Guatemala (9.5%), Nicaragua (2.4%)	PTSD	Customs and Border Protection (CBP) and US Immigration Customs Enforcement (ICE) facilities
Tosif et al., 2023	Retrospective cross-sectional	Australia	*N* = 277, children detained in onshore and offshore detention centres Onshore = 198 Offshore = 79	Median (IQR) = 4.2 (0.7–7.8) Mean and range not reported	Female = 132 (48%) Other genders not reported	Afghanistan (3%), Iran (42%), Bangladesh (< 1%), Cyprus (1%), India (4%), Iraq (4%), Lebanon (2%), Malaysia (7%), Myanmar (1%), Nepal (1%), Pakistan (1%), Sri Lanka (6%), Syria (1%), Thailand (1%), Yemen (1%)	Mental and developmental disorders	Onshore detention – Australian mainland/territories Offshore detention – Nauru or Manus Island
Zayas et al., 2024	Cross sectional	United States	*N* = 81, Central American children previously detained at US-Mexico border	Mean (SD) = 10.88 (2.28) Range = 7–15	Female = 42 (50.6%) Other genders not reported	Honduras (61.5%), El Salvador (10.8%), Guatemala (27.7%)	Emotional and behavioural problems	Detained at US-Mexico border
Zwi et al., 2018	Cross sectional	Australia	*N* = 86 Detainees = 48, asylum-seeking children arrived by boat and detained since arrival Comparison group = 38, newly arrived refugee children settled in non-urban area	Range = 4–15 Mean not reported	Detainees: N/A Community: Male = 18 (48%) Female = 20 (52%)	Detainees: Eastern Mediterranean (50%), South East Asian (18%), Western Pacific (12%), African (< 1%), ‘Stateless’ (19%) Community: South East Asian (29%), African (20%), Eastern Mediterranean (13%)	Emotional and behavioural difficulties	Immigration detention facilities on Christmas Island

a As reported by study authors

b Mental health outcomes which contribute to prevalence data

c Percentages do not total to 100 due to rounding by authors

### Quality assessment

Item-level ratings were used to assess the quality of the included studies (Table 3). Most studies appropriately described the setting/subjects, conducted data analysis with sufficient coverage, used valid methods of identification, conducted a standard measurement of condition, and had an adequate response rate (or appropriately managed low response rates). Eight studies had an adequate sample size; however, this was unclear in six studies. In 12 of studies the appropriateness of statistical analysis was unclear, as they reported on prevalences but did not include confidence intervals. Most of the studies either had inappropriate or unclear sampling methods and frames, due to using convenience sampling rather than probability-based sampling methods.

**Table 3:** JBI critical appraisal checklist for studies reporting prevalence data

Author & Year	Appropriate sample frame	Appropriate sampling method	Adequate sample size	Adequate description of setting/subjects	Data analysis conducted with sufficient coverage	Valid method of identification	Standard measurement of condition	Appropriate statistical analysis	Adequate response rate or management of low response rate
Amarasena et al., 2023	No	No	Unclear	Yes	Yes	Yes	Yes	Unclear	Yes
Bakley et al., 2023	Yes	Yes	Yes	Yes	Yes	Unclear	Yes	Unclear	Yes
Ehntholt et al., 2018	Unclear	No	Unclear	Yes	Yes	Yes	Yes	Unclear	Yes
MacLean et al., 2019	No	No	Yes	Unclear	Yes	Yes	Yes	Unclear	Yes
Min et al., 2020	No	No	Unclear	Yes	Yes	Yes	Yes	Unclear	Unclear
Paloma et al., 2025	No	No	Yes	Yes	Yes	Unclear	Yes	Unclear	Yes
Patler et al., 2025	No	No	Yes	Yes	Yes	Unclear	Yes	Unclear	Yes
Saadi et al., 2022	Yes	Yes	Yes	Yes	Yes	Unclear	Yes	Unclear	Yes
Saadi et al., 2025	Unclear	No	Yes	Yes	Yes	Yes	Yes	Yes	Yes
Santos et al., 2018	Yes	Yes	Yes	Yes	Yes	Yes	Yes	Unclear	Not applicable
Sidamon-Eristoff et al., 2022	No	No	Unclear	Yes	Yes	Yes	Yes	Unclear	Unclear
Tosif et al., 2023	No	No	Yes	Yes	Yes	Yes	Yes	Yes	Not applicable
Zayas et al., 2024	Unclear	No	Unclear	Yes	Yes	Yes	Yes	Unclear	Yes
Zwi et al., 2018	No	No	Unclear	Yes	Yes	Yes	Yes	Unclear	Yes

### Adults

A total of 1685 adult participants were included across the six included studies [48, 49, 52–54, 57]. These studies each reported on the number of male participants, who comprised 89.2% of the adult sample. The reporting of other genders was inconsistent, with three including data on the number of female participants. The age range of participants was only reported in one study; however all studies reported on the mean age of participants, which ranged from 30.9 years to 40.3 years. Five of the studies had cross-sectional designs [48, 49, 52–54], and one had a retrospective observational design [57]. All five cross-sectional studies used self-report instruments to assess mental health outcomes. The retrospective observational study reviewed medical charts of detainees to identify diagnoses of mental conditions. The findings have been categorised by mental health outcomes, and key results from each study can be found in Table 4.

**Table 4:** Results from adult studies

Author & Year	Measurement Instruments ^a^	Prevalence Data	Identified Associated Factors	Length of Detention ^b^	Impact of Detention Duration
Bakley et al., 2023	Self-report of previous diagnosis (yes/no)	Lifetime prevalence of 1 or more mental health conditions = 18.5%	Experiencing abuse in detention significantly associated with increased odds of mental health condition (OR = 4.10, *p* = .003)	< 1 month = 32.2% 1–3 months = 7.6% 3–12 months = 11.6% 12 + months = 45.8%	Being detained for > 1 year significantly associated with increased odds of lifetime diagnosis of mental health condition (OR = 2.81, *p* = .006)
Paloma et al., 2025	Hopkins Symptom Checklist-25, self-report of self-harm (yes/no)	HSCL-25 scores above clinical cutoff ^c^ = 60% Self-harm = 19.5%	Institutional decency (*p* < .01) and relationship with officers (*p* < .05) significantly associated with mental health	0–7 days = 22 7–14 days = 22 15–30 days = 23 31 + days = 20	No analyses conducted
Patler et al., 2025	Self-report of previous diagnosis	At least one disorder = 56.7% Depression = 86.1% PTSD = 56.5% Schizophrenia or bipolar = 15.7% Other condition = 31.3%	Mental illness associated with higher odds of experiencing solitary confinement, interruption of care, and difficulty accessing mental health services in detention	< 6 months = 92 (45.3%) 6 - <12 months = 56 (27.6%) 12 + months = 55 (27.1%)	No analyses were conducted
Saadi et al., 2022	Self-report of previous diagnosis (yes/no)	Diagnosis of mental health condition = 16.43%	Greater number of confinement conditions predicted higher probabilities of mental disorder diagnosis	Mean months detained (SD) = 8.70 (4.68) Range = 3.29–55.53	No analyses conducted
Saadi et al., 2025	Kessler-6-Item (score 13 + indicating mental illness), Primary- Care-PTSD-5 screen	K6 score 13 + = 29.5% PTSD = 48%	N/A	< 6 months = 92 (46%) > 6 months = 108 (54%)	People detained for 6 + months had significantly higher likelihood of mental illness (*p* < .001), and PTSD (*p* < .001), Longer detention duration significantly associated with mental illness (OR = 1.11) and PTSD (OR = 1.11)
Santos et al., 2018	International Statistical Classification of Diseases and Related Health Problems 10th Revision (ICD-10) diagnostic framework	At least one disorder = 28.78% Neurotic, stress-related, and somatoform disorders = 17.67% Mood disorders = 7.32% Mental and behavioural disorders due to psychoactive substance use = 10.60% Personality disorders = 0.76% Schizophrenia, schizotypal, and delusional disorders = 1.01% PTSD = 1.26%	Females were more prone to develop a psychiatric disorder compared to men (χ2 = 7.017; *p* < .05)	No individual data reported, 43% released after 60 days	No analyses conducted

a Instruments used for prevalence data

b As reported by study authors

c Clinical cutoff score = 1.75+

#### Measures of overall psychiatric morbidity

Five adult studies included prevalence data of overall psychiatric morbidity [48, 52–54, 57], however the measures used differed. Two studies [48, 53], asked participants if they had ever received a diagnosis of a mental health disorder, yielding a binary yes/no response. Similarly, one study [52] enquired about previous diagnoses, but asked about a specific range of disorders and calculated the prevalence of having at least one disorder. Another study [57] calculated lifetime prevalence based on detainee’s past medical charts. Finally, one study [54] used a dichotomised measure of mental illness using the Kessler-6 assessment, using a cut-off score of 13 or higher to indicate probable mental illness.

Across all but one of the studies, the prevalence rate of psychiatric morbidity had a fairly narrow range - from 16.4% to 29.5%. One study however reported a substantially higher prevalence of 56.7% [52]. Factors associated with an increased likelihood of experiencing mental illness included experiencing abuse in detention, having a higher number of confinement conditions (e.g. difficulty accessing family visitation, harassment, difficulty accessing psychological services), and being female [48, 53, 57]. Furthermore, individuals with mental health difficulties were more likely to experience solitary confinement, difficulty accessing psychiatric care, and interruption of psychological and medical care [52].

#### Anxiety and mood disorders

Outcomes relating to anxiety and mood disorders were presented in three studies [49, 52, 57], although outcome definitions and measurement approaches varied. One study [57] conducted a chart review to determine the documented prevalence of ICD-10 diagnosed mental disorders and found a 7.3% prevalence of mood disorders. This is notably lower than the estimates from the other studies which used self-report measures. One study [52] found that 86.1% of participants reported having been diagnosed with depression. Another study [49] utilised the Hopkins Symptom Checklist (HSCL-25), a validated self-report scale measuring symptoms of anxiety and depression. They found that 69% of participants had scores above the clinical cut-off for combined depression and anxiety subscales. Disaggregated prevalence data for symptoms of each disorder was not presented. Notably, 71% of participants reported that their symptoms began during detention [49]. The same study also reported that 19.5% of participants had attempted self-harm during detention. Those who self-harmed had a significantly higher severity of anxiety and depression symptoms. In addition, perceived institutional decency and quality of detainees’ relationships with staff were independently associated with mental health.

#### Post-traumatic stress disorder

Three of the included studies assessed the prevalence of post-traumatic stress disorder (PTSD) [52, 54, 57]. Of these, two implemented self-report measures, and one retrospectively reviewed medical charts made during detention. Prevalence estimates differed considerably between the self-report studies and the chart review study. One of the self-report studies [52] asked participants if they had received a previous PTSD diagnosis, to which 56.5% responded affirmatively. The other used the self-report Primary Care PTSD-5 Screen [54]; 48% of participants were identified as likely meeting the criteria for PTSD. In contrast, the chart review yielded a much lower estimate of 1.26% [57].

#### Schizophrenia spectrum and other psychotic disorders

Two studies reported prevalence estimates for schizophrenia and other psychotic disorders among detainees [52, 57]. One study [57] used medical chart reviews to estimate the combined prevalence of schizophrenia, schizotypal, and delusional disorders, at 1.01%. In contrast, the other study [52] used a self-report measure asking whether participants had been diagnosed with either schizophrenia or bipolar disorder, yielding a combined prevalence of 15.7%. However, the latter study did not provide separate estimates for each condition, limiting comparability.

#### Impact of detention duration

In addition to prevalence data, two studies examined associations between detention duration and mental health outcomes [48, 54]. Both studies found significant relationships between longer lengths of detention and the presence of mental health disorders in detainees. The first reported that detention lasting 12 months or more was significantly associated with increased odds of a mental health diagnosis (OR = 2.81) [48]. The second study [54] identified a higher likelihood of both the presence of probable mental illness (scores of 13 or higher on the Kessler-6 assessment) and PTSD among those detained for more than six months. In a sensitivity analysis using a continuous measure of detention duration, they further demonstrated that each additional unit of time in detention was associated with increased odds of mental illness (OR = 1.11), suggesting a dose-response relationship.

### Children and adolescents

Eight studies included samples who were detained as children or adolescents [50, 51, 55, 56, 58–61], with a total of 1092 participants. The age range of participants was three months to 22 years, and the range of mean ages (reported in six studies) was 6.79 years to 19.10 years. A study with participants aged 18 years to 22 years - in addition to adolescents - was included within this category as these individuals were detained when they were minors [58]. All studies were cross-sectional; however, one retrospectively analysed cross-sectional data [60]. The reporting of gender was inconsistent throughout the included studies, with 12.2% of the total sample being male and 25.3% being female, however the gender of 62.5% of participants was not reported. In five studies, parent-report instruments were used to measure child mental health outcomes [50, 51, 55, 56, 61], of which three were completed post-release from detention. Three studies reported that children/adolescents were detained with parents [50, 51, 61], whereas one study included participants who were unaccompanied during detention [58]. The remaining four studies included both participants that had experienced parental separation, and those who had not. The results have been categorised based on mental health outcomes, and the key results from the eight child/adolescent studies can be found in Table 5.

**Table 5:** Results from child & adolescent studies

Author & Year	Measurement Instruments ^a^	Mental Health Prevalence Data	Factors Associated with Mental Health	Length of Detention ^b^	Impact of Detention Duration
Amarasena et al., 2023	Health assessments and clinical judgement of clinicians at first assessment	Symptoms: Low mood (47%), suicidal ideation/attempt or self-harm (45%), anxiety (39%), behavioural (40%), language delay (40%), suspected autism (5%) Diagnoses: Diagnosed condition (44%), pervasive refusal syndrome (15%), PTSD (13%), depression (13%), neurodevelopmental diagnosis (35%)	Significantly increased odds of mental health concern if school aged or older, witnessed trauma, had exposure to 4 + adverse childhood experiences or were from Eastern Mediterranean (all non-significant when controlling for covariates)	0–1 year 11 months = 21% 2–3 years 11 months = 15% 4 + years = 52%	Significantly increased odds of mental concern if held on Nauru of 1 + year (OR = 7.92), odds become non-significant after controlling for covariates
Ehntholt et al., 2018	Structured Clinical Interview for DSM-IV depression and PTSD modules (SCID-VI), Reactions of Adolescents to Traumatic Stress Questionnaire (RATS)	MDD = 9% PTSD = 34% Comorbid MDD and PTSD = 46% Very high RATS score = 80%	N/A	Mean days (SD) = 22.8 (21) Range = 4–92	No analyses conducted
MacLean et al., 2019	Strengths and Difficulties Questionnaire (SDQ), University of California Los Angeles PTSD Reaction Index (UCLA PTSD-RI)	Abnormal SDQ scores: Total difficulties (10%), emotional problems (32%), peer problems (14%), conduct problems (8%), hyperactivity (8%), prosocial (1%) PTSD-RI: 4 symptoms ^c^ (17%), 3 symptoms (18%), 2 symptoms (19%) intrusion (52%), avoidance (57%), negative alterations in mood/cognition (42%), arousal (18%)	4–8 year olds = higher rates of conduct problems, hyperactivity, and total difficulties than older children Children forcibly separated from mothers had significantly more emotional and total difficulties	1–9 days = 61% 10–19 days = 35% 20–29 days = 2% 30–39 days = 1% 40–44 days = 1%	No analyses conducted
Min et al., 2020	Strengths and Difficulties Questionnaire (SDQ)	Borderline/abnormal SDQ scores ^d^: Total difficulties (59.5%), emotional problems (78.6%), peer problems (57.1%), conduct problems (50%), hyperactivity (57.1%)	Mothers PTSD significantly related to child emotional problems, and mothers depression associated with child peer problems	Mean days (SD) = 35.62 (24.82) Range = 3–86	Time spent in family detention centre not significantly related to child behavioural symptoms
Sidamon-Eristoff et al., 2022	University of California Los Angeles PTSD Reaction Index (UCLA PTSD-RI)	4 symptoms ^c^ (6.49%), 3 symptoms (5.19%), 2 symptoms (18.18%), intrusion (37.66%), avoidance (22.27%), negative alterations in mood/cognition (22.08%), arousal (18.18%)	PTSD severity positively correlated with rates of pre-migration trauma (p = < 0.001)	Mean days (SD) = 7.31 (7.15) Range = 1–26	PTSD severity not significantly correlated with length of detention (*p* = .233)
Tosif et al., 2023	Clinical diagnoses by child psychiatrist, paediatrician, or psychologist, or if recorded symptoms met DSM-5 criteria	Onshore sample: PTSD (22%), depression (24%), anxiety (36%), behaviour disorder (37%), attachment disorders (15%), self-harm (4%), intellectual disability (12%), autism (11%) Offshore sample: PTSD (49%), depression (54%), anxiety (63%), behaviour disorder (49%), attachment disorders (48%), self-harm (27%), intellectual disability (4%), autism (6%)	Children detained on Nauru has significantly higher prevalence of all mental health concerns compared to those held in Australian detention centres	Median months (IQR): Onshore sample = 7 (4–16) Offshore sample = 51 (29–60)	No analyses conducted
Zayas et al., 2024	The Behaviour Assessment System-3 (BASC-3)	Clinically significant levels = 1 + BASC-3 dimension ^e^ (16%), anxiety (12.5%) High risk ^f^ = anxiety (13.58%) depression (7.40%), low self-esteem (9.87%), social stress (13.58%), low self-reliance (16.04%), locus of control 7.40%), sense of inadequacy (9.87%), atypicality (11,11%)	Children of families who experienced abuse in detention = higher mean anxiety and depression scores Children who experienced parental separation = higher mean anxiety scores	Mean days (SD) = 14.48 (22.33) Less than 10 = 50.60% 20 + = 19.28% 100 + = 3 families	No analyses conducted
Zwi et al., 2017	Strengths and Difficulties Questionnaire (SDQ)	Abnormal SDQ scores: Total difficulties (25%), emotional problems (75%), peer problems (25%), conduct problems (39.5%), hyperactivity (43.8%), prosocial (25%)	N/A	Data available = 60% of sample Mean days = 221 Range = 90–390 > 100 = 6 > 200 = 22 > 300 = 1	No significant correlation between total difficulties scores and days in detention (*p* = .98)

a Measurement tools contributing to prevalence data

b As reported by study authors

c 4 symptoms = probable PTSD

d Study combined borderline and abnormal score prevalences

e Clinically significant levels = t score of 70 or higher

f High risk = t score of 60 or higher

#### Emotional and behavioural difficulties

The parent-report Strengths and Difficulties Questionnaire (SDQ) was used in three studies to assess child emotional and behavioural difficulties [50, 51, 61]. The SDQ includes one strengths subscale (prosocial behaviour) and four difficulties subscales (emotional symptoms, peer problems, conduct problems, and hyperactivity). Each subscale, along with the total difficulties score (sum of the four difficulties subscales), is classified as normal, borderline, or abnormal, with abnormal scores indicating significant difficulties requiring professional support. Only two studies reported data for prosocial behaviour [50, 61], however all studies reported on the difficulties subscales.

Two studies reported the prevalence of children scoring in the abnormal range [50, 61], whereas one study applied a broader cut-off combining borderline and abnormal scores [51]. Reported prevalence of abnormal total difficulties ranged from 10% to 25% in the studies using abnormal-only thresholds. Emotional symptoms consistently showed the highest prevalence (32–75%), while conduct problems had the lowest (8–39.5%). The study using the combined borderline and abnormal cut-off reported much higher overall rates, with emotional symptoms again most frequent (78.6%) and conduct problems lowest (50%).

Among studies reporting abnormal-only prevalence, one [61] observed higher prevalences across all difficulties subscales and total difficulties than the other [50]. The same study demonstrated that detained children had significantly higher abnormal scores in all subscales (except prosocial behaviour) than a community-based migrant sample. Age-related differences were identified in one study [50], with younger children (four to eight years old) showing higher abnormal scores for conduct problems, hyperactivity, and total difficulties, than older children. Maternal factors were also associated with SDQ outcomes: maternal separation during detention was linked to elevated emotional symptoms and total difficulties [50]. In the study using the combined cut-off [51], maternal PTSD was associated with child emotional symptoms, maternal depression was associated with child peer problems, and maternal exposure to violence during their migration journey was associated with higher conduct problems.

#### Anxiety and depression

Four studies reported on the prevalences of depression and/or anxiety in child detainees [56, 58–60], though the specific outcomes and measurement tools differed. One study measured diagnoses of major depressive disorder (MDD) [58], one reported on diagnoses of depression and anxiety [60], one reported on anxiety symptoms and depression diagnoses [59], and one on children at high risk of both disorders as well as those who met the clinical criteria for anxiety [56]. In detained children, the prevalence of clinical diagnoses of depression ranged from 13% to 55% [58–60]. Of these studies, one reported both MDD prevalence (9%) and comorbid MDD and PTSD prevalence (46%), which totalled to a 55% prevalence of MDD [58]. Anxiety features not meeting a diagnostic criterion (significant symptoms and/or individuals identified as high risk) ranged from 13.58% to 39% [56, 59]. Anxiety features meeting a diagnostic criterion ranged from 12.5% to 63% across two studies [56, 60]. One of these studies reported on anxiety and depression diagnoses in children detained in onshore and offshore detention in Australia, revealing that children in offshore detention settings had significantly higher rates of both disorders compared to children detained onshore [60]. The second study noted that children of families that had experienced psychological or verbal abuse in detention (reported by 17% of children) had significantly higher mean anxiety and depression scores, and children who experienced parental separation had higher mean anxiety scores [56].

#### Post-traumatic stress disorder

PTSD was the most frequently investigated mental health diagnosis in child samples, with five studies reporting prevalence data [50, 55, 58–60]. Three studies used clinical diagnostic measures [58–60]. Reported prevalences of PTSD diagnoses among child detainees ranged from 13% to 80%. The highest prevalence (80%) was reported in a study of adolescents and young people who had previously been detained unlawfully as unaccompanied minors (detained under the premise of being adults) [58]. The prevalence result combines PTSD-only (34%) and comorbid PTSD-MDD (46%) diagnoses. Using the Reactions of Adolescents to Traumatic Stress Questionnaire, the same study found 80% of the sample to have very high scores, indicating severe PTSD symptoms. One study reported a higher prevalence of PTSD in those detained offshore (49%) compared to onshore (22%) in Australia [60].

The UCLA Child/Adolescent PTSD Reaction Index Scale for DSM-5 (UCLA PTSD-RI) was used in two studies; it assesses the severity of the four DSM-5 PTSD symptom clusters (intrusion, avoidance, negative alterations in cognition/mood, and arousal/reactivity), with severe scores in all four indicating probable PTSD. One study [50] administered the UCLA PTSD-RI to children detained by US Immigration and Customs Enforcement (ICE) at the time of assessment; and the other study assessed children previously detained by ICE or Customs and Border Protection (CBP) [55]. The proportion with severe scores in all four clusters was 17% [50] and 6.49% [55]. One study also found that 18% of children had severe scores in three clusters [50], and the most prevalent severe symptom found in this study was avoidance (57%). In the other study, intrusion was the most prevalent severe symptom (37.66%) [55]. Increased arousal/reactivity was the least prevalent severe symptom in both (18%).

#### Neurodevelopmental disorders

Two studies reported prevalence data on neurodevelopmental disorders [59, 60], with variation according to symptom-based versus diagnostic measures and broad versus specific diagnostic categories. One study included participants in offshore Australian detention [59], and the other compared offshore and onshore detainees [60]. Both reported on diagnoses of neurodevelopmental disorders, however one study [59], presented data on the prevalence of these disorders as a broad group, which was 35%. This study also reported on symptoms of neurodevelopmental concerns, including language delay, at 40%, and suspected autism spectrum disorder (ASD), at 5%. The other study presented intellectual disabilities and ASD separately [60]. In this study, the prevalence of intellectual disabilities ranged from 4% (offshore) to 12% (onshore), and ASD prevalences ranged from 6% (offshore) to 11% (onshore), indicating a higher prevalence of neurodevelopmental disorders in children detained onshore.

#### Impact of detention duration

Of the nine studies with child participants, four analysed the relationship between the length of time in detention and mental health outcomes [51, 55, 59, 61]. The length of detention for participants in these studies ranged from less than one day [55] to over four years [59]. Three studies reported the mean number of days in detention, which ranged from 7.31 to 390 days. Only one study reported a significant relationship between duration of detention and mental health, finding that being detained for one year or more resulted in higher odds of having a mental health condition (OR = 7.92) [59]. However, this relationship became non-significant after controlling for covariates (which were not specified). PTSD severity was not significantly correlated with time spent in detention [55]. Two studies reported on the relationship between time in detention and emotional and behavioural difficulties in children [51, 61], both concluding that there was no significant correlation with SDQ scores and the length of detention.

## Discussion

### Prevalence of mental disorders and symptoms

This systematic review included 14 studies which investigated the impact of immigration detention on the prevalence of mental health diagnoses and symptoms. There was consistent evidence that various mental health conditions and symptoms were highly prevalent in immigration detainees. These findings were identified irrespective of country of detention and were evident in both adult and child/adolescent detainees. Adult samples demonstrated high prevalences of psychiatric morbidity, especially PTSD, mood disorders, and anxiety. This aligns with both the findings from the 2018 systematic review [42], as well as previous literature, which identify depression, anxiety, and PTSD as the most common mental health conditions in detainees [36, 37]. One study of adult detainees conducted a retrospective medical chart review and reported notably lower prevalences of all conditions [57]. This may be representative of limited psychiatric assessments in detention leading to under-reporting, which has been identified in past literature [62].

Several detention-related factors were associated with mental illnesses in adults, including experiencing abuse, conditions of confinement, staff relationships, and institutional decency. This highlights that various aspects of the detention process contribute to creating an invalidating environment influencing emotional distress [63]. Furthermore, detainees with mental illnesses were more likely to face institutional difficulties such as solitary confinement and difficulty accessing mental health services. Only one study [57] reported on sub-group vulnerabilities to mental illness, concluding that female detainees had a higher likelihood of psychiatric diagnoses.

Child detainees demonstrated similarly high prevalences of PTSD, depression, and anxiety, which is in line with previous findings [64, 65]. Children had particularly high prevalences of PTSD, regardless of the detention location. The high prevalence of PTSD in both child and adult detainees may highlight that the process of detention triggers re-traumatisation in asylum-seekers [41]. Half of the child studies took place in USA detention, where there is evidence of limited access to appropriate paediatric mental health screening and case management [66]. In studies exploring Australian detention, children held in offshore centres had significantly higher prevalences of all mental disorders than those in onshore centres. The process of offshore detention is known for being arbitrary and allowing systemic human rights abuses [67, 68], which may contribute to these higher prevalences. In addition to mental disorders, there was evidence of neurodevelopmental conditions and symptoms in child detainees. The prevalence of these conditions was slightly higher in these detainees compared to data presented in the 2019 Global Burden of Disease study, which ranged from 4.1% to 7.0% [69]. There is minimal research into neurodevelopmental disorders in child detainees specifically, although high prevalences have been identified in both asylum-seeking children [70], and children of refugee parents [71].

There were also significant emotional and behavioural difficulties in child detainees, with emotional symptoms consistently being the most prevalent. These emotional symptoms include depression, somatic presentations, fear and worry, and anxiety [72]. Previous research using the Strengths and Difficulties Questionnaire in child detainees found comparable results [73]. Whilst these studies did not look specifically at anxiety and depression diagnoses, the high prevalence of emotional symptoms could be indicative of the children having these disorders or being at risk of developing them. One study compared child detainees to a community sample and found detained children to have higher emotional and behavioural difficulties in all areas. Various maternal factors were associated with emotional and behavioural difficulties in children, including maternal separation, maternal mental health, and maternal exposure to violence during the migration journey. Migration-related family separation has previously been linked to increased depression, anxiety, and emotional and behavioural problems in children [74]. Furthermore, longitudinal data supports the findings that maternal difficulties are positively correlated with child emotional and behavioural problems [75]. These findings highlight that harms observed in detained children are not solely due to individual vulnerabilities, but also to detention environments which create conditions that undermine maternal mental wellbeing and disrupt parent-child relationships.

### Impact of detention duration

In studies of adult detainees, there was consistent evidence of a significant relationship between the length of time in detention and having a diagnosed mental illness. This was found regardless of country of detention. These findings align with previous literature, including the 2018 review [40, 42]. There was evidence of a possible dose-response relationship between detention duration and mental illness, with a unit increase in detention length associated with an increase in symptoms of psychiatric morbidity and PTSD, respectively. This highlights that adults may be vulnerable to cumulative exposure to traumatic negative events, which has been linked with psychological disorders, including PTSD [76, 77].

This review did not find evidence of a relationship between length of detention and mental illness in child samples, with high rates of psychiatric morbidity reported even in the contexts of brief detention. Similar findings were highlighted in the 2018 review [42], which also noted limited evidence of duration predicting child outcomes. These patterns demonstrate that children are acutely vulnerable to mental health problems in detention, independent of exposure length, as noted in earlier findings [73]. This is consistent with developmental research showing that children’s reliance on stable caregiver relationships makes them particularly sensitive to disruptions in parental availability [78]. Detention frequently undermines parental roles, leaving children without consistent sources of comfort and security [79]. At the same time, environments that strip children of control, predictability, and agency intensify stress responses and foster helplessness, reducing resilience and wellbeing [80–82]. Together, the dual impact of disrupted caregiving and loss of agency provides a compelling explanation for why children exhibit such prominent levels of psychiatric morbidity in detention, even over relatively short periods.

### Limitations of included studies

All but one of the included studies used cross-sectional designs, which indicates that there remains a lack of longitudinal evidence exploring the long-term impact of detention on mental health. The nature of cross-sectional data collection means a causal relationship between detention and adverse mental effects cannot be determined from the existing evidence. The quality appraisal assessment conducted (Table 2) revealed that most studies had inappropriate sampling frames and sampling methods. This is due to most implementing non-probability convenience sampling of participants. However, there are systemic barriers to conducting research in detention using probability-sampling, including physical and security access restrictions, and legislative challenges [83]. In six studies, it was unclear if the sample size was adequate; this was due to the unavailability of sample size calculations in the study methods sections. Furthermore, the quality appraisal indicated an unclear risk of bias in the statistical analysis across most studies, attributable to the absence of confidence intervals for reported prevalence estimates. This limits the ability to assess the precision and uncertainty of the reported values; hence the results of the review should be interpreted as descriptive.

### Strengths and limitations of review

This review involved a comprehensive search of six databases, using broadly inclusive search terms to capture all relevant papers. For example, terms relating to reception centres and temporary accommodation were included despite the review not looking at these settings specifically. Furthermore, we included studies that reported on mental health symptoms as well as diagnoses, as access to validated diagnostic tools may be limited in detention environments [62], and limiting our inclusion criteria to diagnoses would have excluded informative symptom-based data.

The limitations of the current review include restricting the results to papers written in English. This may have excluded valuable evidence produced in other languages, as many detention centres are in non-English speaking countries (e.g. Yemen, Libya). Moreover, focusing on studies reporting prevalence data resulted in studies which reported on mental health data using alternative means (e.g. mean scores, regression analyses) being excluded from this review. Regarding length of detention, there were large variations between and within studies in how long participants were detained. For example, some participants spent only one day in detention, whereas others were detained for several years. This limits the comparability of participants experiences of detention, and its subsequent impact on participants mental wellbeing. Finally, whilst the use of the JBI Critical Appraisal Checklist for Studies Reporting Prevalence Data was well suited for the included studies, it did not allow for definitive values of quality to be assigned to each study. Therefore, it was not possible to identify if findings were consistent across studies of higher methodological quality.

### Advances since the 2018 review

The findings of this review align with those of von Werthern and colleagues [42], noting high prevalences of mental disorders and symptoms among both adults and children in detention, particularly post-traumatic stress disorder, depression, and anxiety. Consistent with the earlier review, the present synthesis found evidence that longer durations of detention are associated with greater mental health difficulties in adults, although the evidence base for children remains limited. Importantly, this review extends the 2018 findings by incorporating studies with larger sample sizes, thereby strengthening confidence in the overall conclusions. In addition, it contributes novel insights by synthesising literature on emotional and behavioural symptoms in child detainees, and by identifying associations between detention-related experiences and negative psychological outcomes.

### Conclusions and recommendations

This review demonstrates that there is consistent evidence that immigration detention is associated with high psychiatric morbidity in both adults and children. The most common symptoms and diagnoses were those of PTSD, depression, and anxiety. Children also showed high rates of emotional, behavioural, and neurodevelopmental concerns. Detention conditions and institutional factors, such as abuse, confinement, and experiencing offshore detention, are associated with an increased risk of mental illness, highlighting the presence of systemic and harmful attributes of the detention process. Furthermore, there is now clear and consistent evidence that in adults, the length of time spent in detention is associated with increased mental health concerns. This relationship is not demonstrated in children, reflecting the harmful effects of even brief periods of detention.

We recommend that based on these findings, an upper limit should be applied to adult detention duration in countries without such restrictions. Furthermore, the conditions of detention environments should be examined, with an emphasis on eliminating harmful practises (e.g. separation of families, verbal and physical abuse, poor access to health services). Mental-health screening should be conducted prior to detention, to identify those at risk or those with disorders. Evidence-based psychological support accessible for individuals held in detention. Governments should also consider alternatives to immigration detention, such as community-based arrangements. The detention of children should be abolished given the clear evidence of harm and based on existing UNHCR guidelines. Our recommendations for future research include conducting longitudinal studies, to further develop the understanding of whether the relationship between detention and mental illness is causal in nature, and to identify possible protective factors which may aid immigration detainees.

## Data Availability

No datasets were generated or analysed during the current study.

## Abbreviations

UNHCR: United Nations High Commissioner for Refugees
UK: United Kingdom
USA: United States of America
PTE’s: Personal traumatic events
PTSD: Post-traumatic stress disorder
SDQ: Strengths and Difficulties Questionnaire
MDD: Major Depressive Disorder
HSCL-25: Hopkins Symptom Checklist-25
UCLA PTSD-RI: UCLA PTSD Reaction Index for DSM-5
ICE: Immigration and Customs Enforcement
CBP: Customs and Border Protection
ASD: Autism Spectrum Disorder
